# Supplementary material for: High‐Entropy Alloys and Their Affinity with Hydrogen: From Cantor to Platinum Group Elements Alloys
Source: Adv Sci (Weinh). 2024 Jun 18;11(31):2401741. doi: 10.1002/advs.202401741 (PMC11336920; doi:10.1002/advs.202401741)
Supplement: Supplementary file 1 — Supporting Information [file ADVS-11-2401741-s001.pdf]

ADVANCED SCIENCE
Open Access

Supporting Information

for *Adv. Sci.*, DOI 10.1002/advs.202401741

High-Entropy Alloys and Their Affinity with Hydrogen: From Cantor to Platinum Group Elements Alloys

Konstantin Glazyrin, Kristina Spektor, Maxim Bykov, Weiwei Dong, Ji-Hun Yu Yu, Sangsun Yang, Jai-Sung Lee Lee, Sergiy V. Divinski, Michael Hanfland and Kirill V. Yusenko*

SUPPLEMENTARY

High-Entropy Alloys and their Affinity with Hydrogen: from Cantor to Platinum Group Elements Alloys.

K. Glazyrin,[a] K. Spektor,[a] M. Bykov,[b] W. Dong,[a] J.-H. Yu,[c] S. Yang,[c] J.-S. Lee,[d] S. Divinski,[e] M. Hanfland,[f] K. V. Yusenko[g]

[a] Photon Sciences, Deutsches Elektronen-Synchrotron, Notkestr. 85, 22607 Hamburg, Germany, [b] Institute of Inorganic Chemistry, University of Cologne, Cologne, Germany, [c] Powder Materials Division, Korea Institute of Materials Science, 51508 Changwon, South Korea, [d] Department of Materials Science and Chemical Engineering, Hanyang University, 15588 Ansan, South Korea, [e] Institute of Materials Physics, University of Münster, D-48149 Münster, Germany, [f] ESRF – The European Synchrotron, 71 Av. des Martyrs, 38000, Grenoble, France, [g] Bundesanstalt für Materialforschung und –prüfung (BAM), D-12489 Berlin, Germany

1. Compression of industrial alloys *fcc*–A190, *fcc*–TM3 and 316 Steel

Here and below we show some additional information with respect to the industrial alloys. In addition to the study on *fcc*–A190 and *fcc*–TM3 (**Figure S1**) we also conducted a study on conventional 316 Steel purchased from GoodFellow® [1]. From the point of X-ray diffraction, this steel material can be described as two-phase composition: the martensitic (*bcc*) and the austenitic (*fcc*) phases (**Figure S2**).

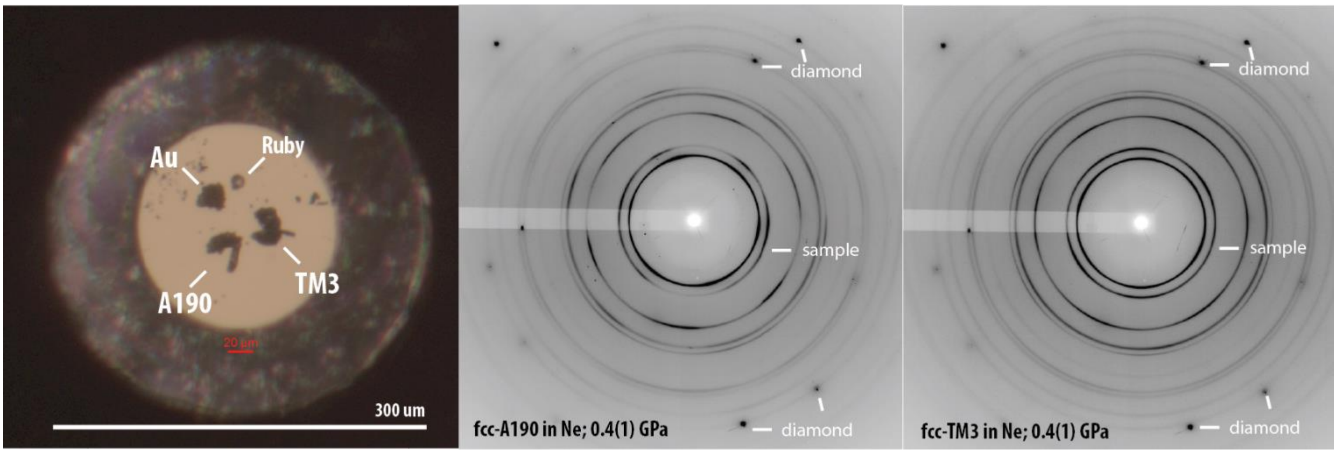

Figure S1 Images of (left) microphotograph of a diamond anvil cell sample chamber prior to hydrogen loading. It shows *fcc*–A190 (Alloy 190), *fcc*–TM3 (ToughMet 3) as well as chips of ruby and gold. Ruby was used for pre-compression after gas-loading and gold was used for pressure determination during compression in H_2. The images to the (right) and in the (middle) show 2D diffraction images of the alloys loaded with Ne. The same images confirm abundant presence of *fcc*-structured phases with preferred orientation of the grains due to manufacturing process.

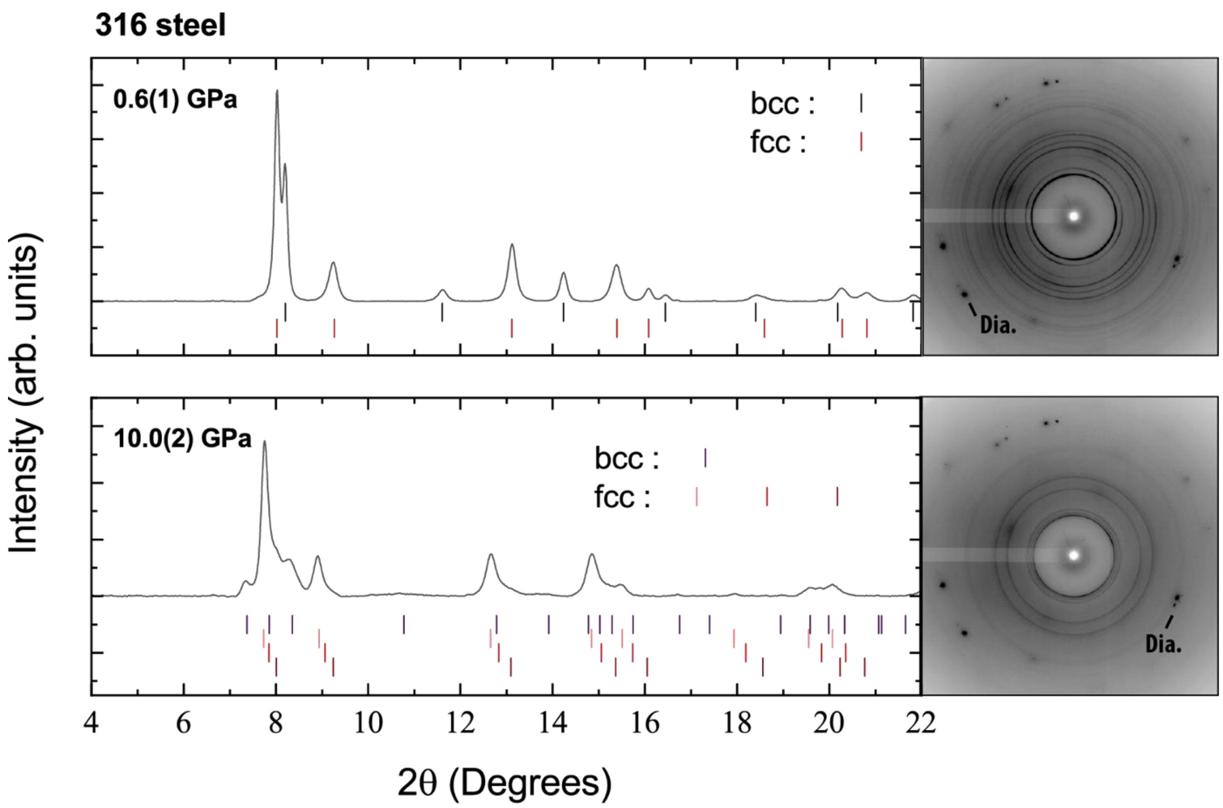

Figure S2 Selected diffractograms for 316 steel compression in H_2 pressure medium. Under compression we see formation of and *hcp*, *fcc* hydrides with different hydrogen content, pressure dependent. Considering the *fcc* phase, we see slow capture of hydrogen, with intermediate hydrogen content evident through the tails of *fcc* phase peak for 10.0(2) GPa diffractogram. We indicate peaks from diamond anvils at the corresponding 2D image.

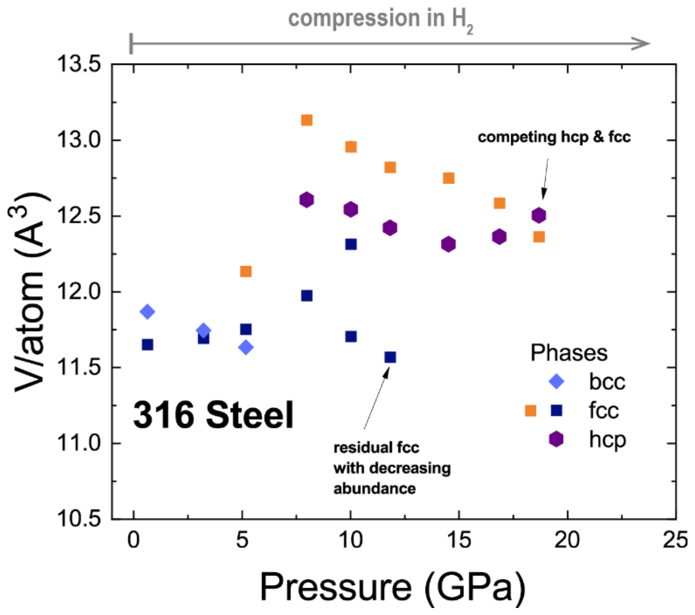

Figure S3 Atomic **v**olume for phases measured upon compression of steel ALSI 316 [1] in hydrogen at room temperature. The *fcc* and *bcc* phases present at ambient are converted to hydrides at relatively low pressure. At pressures above 5 GPa we could not observe *bcc* as it transforms to the *hcp* phase. The positive trend of *fcc* phase volume as a function of pressure range below 10 GPa indicates that this phase starts to pick up hydrogen early.

Considering the data collected from 316 steel we see that hydrogen is captured by the material already at low pressures in contrast to *fcc*–A190. Considering the chemical composition, the steels 304, 316 and 316L steels are not that different. This difference is very important for their mechanical properties after tempering acid resistance (higher for 316 and 316L), but we consider that, although some details will be different, in general they will behave very similar under compression with hydrogen.

2. Compression of *fcc*–PGM and *hcp*–PGM

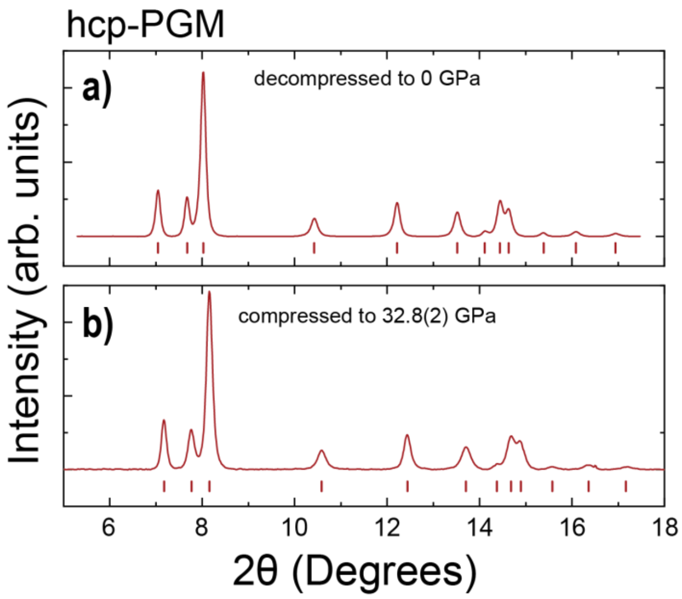

Figure S4 X-ray diffraction patterns collected on *hcp*–PGM (composition $Ir_{0.19(1)}Os_{0.22(1)}Re_{0.21(1)}Rh_{0.20(1)}Ru_{0.19(1)}$). In a) we show data collected after decompression in H_2 pressure transmitting medium (PTM) to ambient pressure while in b) we show diffraction patterns after compression in H_2 PTM with finite amount of hydrogen captured within the crystal lattice of the compound.

3. Compression of Cantor Alloy

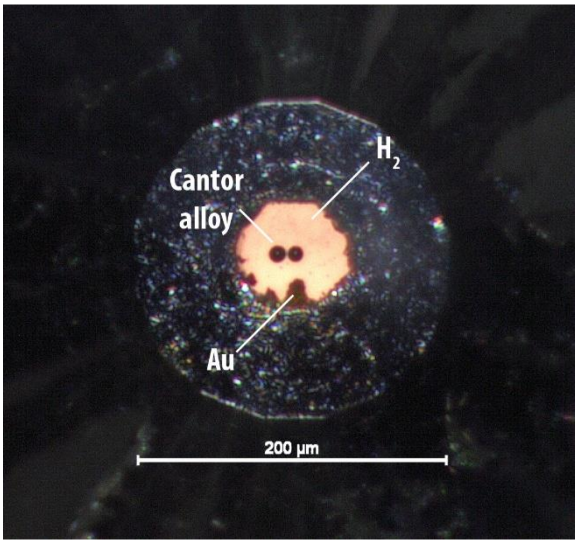

Figure S5 Microphotograph showing small spheres of Cantor alloy loading with hydrogen as pressure-transmitting medium. We used powder Au with sub-micron grain as pressure standard.

References

[1] GoodFellow, *AISI 316 Stainless Steel Alloy Rod*, https://www.goodfellow.com/p/fe24-rd-000140/aisi-316-stainless-steel-alloy-rod.
